# Green solvent-based extraction of *Cannabis sativa* L. by-products: enhancing antioxidant potential and cannabinoid enrichment using MCT oil

**DOI:** 10.1186/s42238-025-00309-4

**Published:** 2025-07-26

**Authors:** Nipaporn Meepun, Ubonta Sommart, Oraporn Bualuang

**Affiliations:** Chemistry Program, Faculty of Science and Technology, Suratthani Rajabhat University, Surat Thani, 84100 Thailand

**Keywords:** Cannabis by-product, Oil base extraction, CBD

## Abstract

**Background:**

With the growing interest in the therapeutic applications of *Cannabis sativa* L., understanding how extraction techniques influence its antioxidant potential and phytochemical composition is essential for optimizing product quality and functionality.

**Methods:**

This study evaluated the impact of various oil-based extraction methods—specifically using medium-chain triglycerides (MCT)—on the antioxidant activity and cannabinoid content of *Cannabis sativa *sugar leaf, leaf, and root. The extraction techniques investigated included ultrasound-assisted extraction (UAE), heated infusion extraction (IE), ultrasound-assisted infusion extraction (UIE), and microwave-assisted extraction (MAE).

**Results:**

Significant differences were observed in the physicochemical and antioxidant properties of the extracted oils. Compared to UAE, the IE, UIE, and MAE methods resulted in 26.4–148.3% higher color intensity. Peroxide values ranged from 0.055 to 0.276 mg O₂ eq/kg, indicating acceptable oxidative stability. Total phenolic content varied between 2.16 and 125.27 mg gallic acid equivalents (GAE)/100 g, with IE of sugar leaf yielding 131.8% higher phenolics than root extracts. Antioxidant assays revealed that IE-extracted sugar leaf oil showed superior activity, with increases of 3.16-fold (DPPH), 0.32-fold (ABTS), 6.18-fold (FRAP), and 3.89-fold (1,10-phenanthroline) compared to UAE. Furthermore, the IE method yielded the highest levels of CBD (0.027 g/100 g dry matter) and THC (0.746 g/100 g dry matter) in sugar leaf extracts.

**Conclusions:**

These findings highlight the sugar leaf as a valuable by-product rich in bioactive compounds. Heated infusion extraction notably enhances the antioxidant and cannabinoid content of cannabis oils, offering potential for nutraceutical and cosmetic applications.

## Introduction

*Cannabis sativa* L. is increasingly recognized not only for its primary psychoactive and therapeutic cannabinoids, such as cannabidiol (CBD) and Δ⁹-tetrahydrocannabinol (THC), but also for a diverse array of secondary metabolites with antioxidant, anti-inflammatory, and potential cosmeceutical properties (Andre et al. 2016; Ahmed et al. 2019). While existing research has predominantly focused on inflorescences, substantial quantities of residual biomass—including sugar leaves, fan leaves, and roots—remain underutilized, despite mounting evidence that these by-products are rich in bioactive constituents such as phenolics, flavonoids, terpenoids, and minor cannabinoids (Isidore et al. 2021). Recent GC–MS-based profiling of wild fruits such as olive, jujube, and common fig revealed a rich diversity of phenolic acids—including both free and conjugated forms—with 2,4-dihydroxybenzoic acid and trans-cinnamic acid as dominant constituents, supporting their potential for nutraceutical development (Ahmad et al. 2021).

In pursuit of sustainable valorization of cannabis cultivation residues, medium-chain triglycerides (MCTs) have emerged as promising green solvents. MCT oils, commonly derived from coconut or palm kernel oil, are biocompatible, food-grade lipids with excellent solubilizing capacity for hydrophobic compounds, including cannabinoids (McClements 2020). Their favorable sensory profile and intrinsic antioxidant properties further support their suitability for nutraceutical and cosmetic applications (Nimbkar et al. 2022).

Advanced extraction technologies such as ultrasound-assisted extraction (UAE), microwave-assisted extraction (MAE), and heated infusion-based approaches have demonstrated enhanced extraction efficiency, selectivity, and compound stability (Kumar et al. 2021; Vinatoru et al. 2017). However, there is limited comparative research evaluating these oil-based methods in the context of cannabis by-products. Specifically, little is known about how different extraction techniques influence the antioxidant capacity, cannabinoid profile, and physicochemical characteristics of oils derived from distinct plant tissues.

This study addresses these gaps by systematically investigating four MCT-based extraction techniques—UAE, heated infusion extraction (IE), ultrasound-assisted infusion extraction (UIE), and MAE—applied to sugar leaves, fan leaves, and roots of *Cannabis sativa*. Key parameters include total phenolic content, free radical scavenging activity (DPPH, ABTS), ferric reducing antioxidant power (FRAP), transition metal chelation capacity, color intensity, and cannabinoid composition (CBD and THC). The findings aim to inform the optimal extraction strategy for maximizing bioactivity and product quality from cannabis by-products, supporting their sustainable application in health and cosmetic industries.

This is the first study to systematically compare multiple MCT-based extraction approaches applied to different *Cannabis sativa* L. tissues, linking extraction efficiency to antioxidant potential and cannabinoid content. This work thus offers a novel perspective on upcycling cannabis biomass using green, lipid-based extraction technologies.

## Materials and methods

### Plant material and botanical description

The plant materials used in this study—including leaves, sugar leaves, and roots of *Cannabis sativa* L.—were obtained from KD Sriwichai Co., Ltd., located in Kanchanadit District, Surat Thani Province, Thailand. The cannabis cultivar, designated ‘KD’, was officially certified by the Department of Agriculture, Thailand, as *Cannabis sativa* L. This variety is registered under Sect. 28 of the Thai Plant Varieties Protection Act B.E. 2518 (1975), as amended by the Plant Varieties Protection Act (No. 2) B.E. 2535 (1992), thereby confirming its legal status and botanical authenticity. Harvesting was conducted between January and May 2023, following standardized cultivation protocols under controlled environmental conditions.

### Sample preparation

Immediately after harvest, the plant materials were dried in a hot-air oven at 50 °C for 24 h to reduce microbial load and stabilize moisture content. Drying was continued until a constant weight was achieved, resulting in a final moisture content of 6.87 g per 100 g dry weight (w/w, dry basis) (Bhatt et al. 2018). This drying temperature was selected based on previous studies demonstrating that thermal degradation of cannabinoids and polyphenols is minimal below 60 °C (Caputo et al. 2022).

The dried materials were then ground into a fine powder using a stainless-steel laboratory grinder to ensure sample homogeneity prior to extraction and stored in airtight, opaque containers at 5 °C until further analysis.

### Oil-based extraction method

**Fig. 1:**
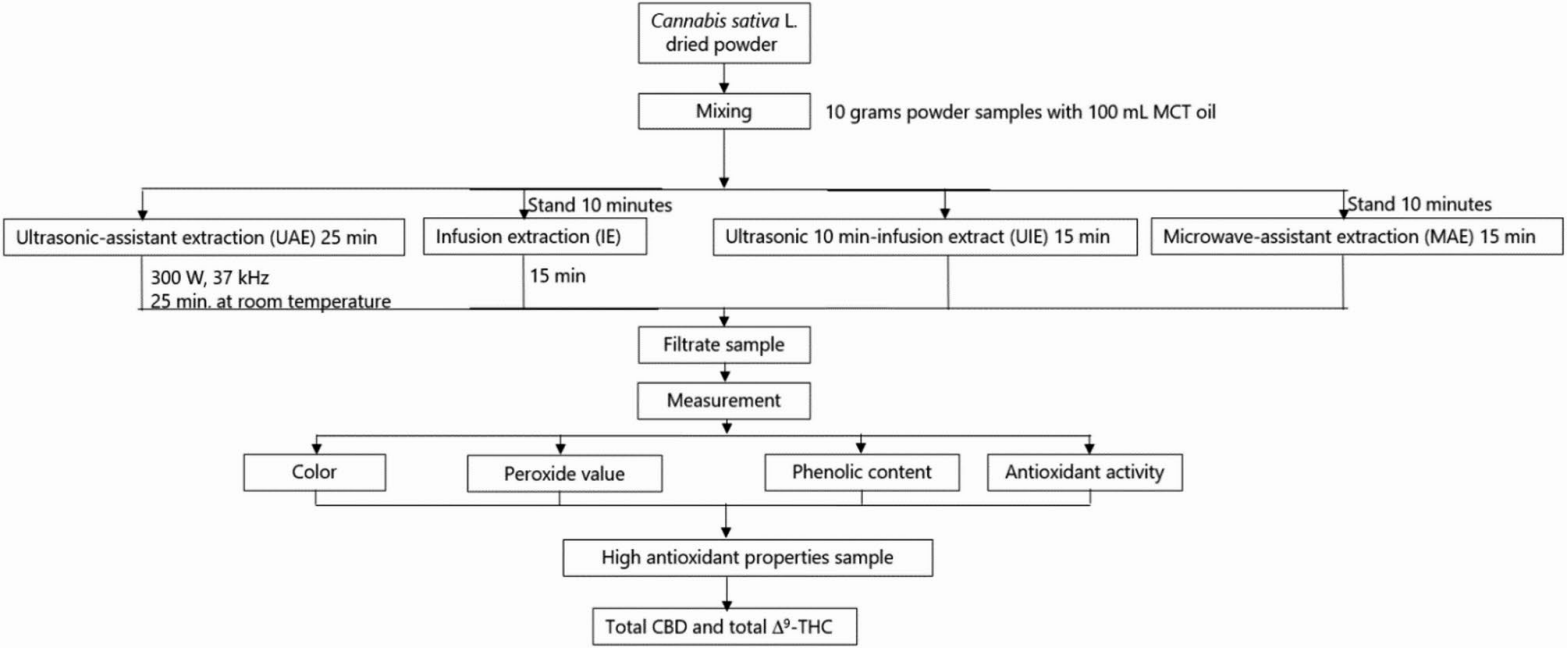
Schematic of extraction process

The oil-based extraction procedures employed in this study are illustrated in Fig. 1.

### Ultrasonic-assisted extraction (UAE)

Ultrasonic-assisted extraction was adapted from the method reported by Vinatoru et al. (2017), with minor modifications. Ten grams of powdered cannabis from each plant part were placed into individual 250 mL Erlenmeyer flasks, and 100 mL of food-grade medium-chain triglyceride (MCT) oil was added. The mixtures were sonicated using an ultrasonic processor (Elmasonic 300 H, Germany) at 37 kHz and 300 W for 25 min at ambient temperature. Ultrasonication promotes cell wall disruption and enhances the release of intracellular compounds such as cannabinoids and phenolics (Chemat et al. 2017). After treatment, the suspensions were filtered under vacuum through Whatman No. 1 filter paper. Extracts were stored in amber bottles at 5 °C until analysis. All extractions were performed in triplicate.

### Infusion extraction (IE)

The infusion method was adapted from techniques used in herbal lipid extraction (Lazar et al. 2021). Briefly, 10.000 g of cannabis powder was mixed with 100 mL of MCT oil in an Erlenmeyer flask and allowed to stand for 10 min to enable initial solvent penetration. The mixture was then transferred to a domestic pressure cooker (Houseworth HW-P004, 2.8 L) and heated for 15 min. The internal temperature was monitored throughout the process. This thermal treatment facilitates the diffusion and solubilization of lipophilic compounds, including cannabinoids (Pavlovic et al. 2018). After cooling, the extract was filtered and stored at 5 °C. All extractions were carried out in triplicate.

### Ultrasonic-infusion extraction (UIE)

The ultrasonic-infusion extraction method was developed by integrating the principles of UAE and IE. Ten grams of cannabis powder were mixed with 100 mL of MCT oil and sonicated for 10 min. The pretreated mixture was then transferred to a pressure cooker and heated for an additional 15 min. This hybrid technique combines mechanical disruption from ultrasound with thermal diffusion, enhancing the extraction efficiency of bioactive compounds. The final extract was filtered and refrigerated at 5 °C until analysis. All experiments were performed in triplicate.

### Microwave-assisted extraction (MAE)

Microwave-assisted extraction was conducted following protocols established by Chemat et al. (2017). A total of 10.000 g of cannabis powder and 100 mL of MCT oil were combined in an Erlenmeyer flask. The mixture was subjected to microwave irradiation using a domestic microwave oven (Samsung, Thailand) operating at 2,450 MHz and 600 W. To prevent overheating and preserve heat-sensitive compounds, power was applied intermittently—1 min on, followed by 5 min off—until a total irradiation time of 15 min was reached. Internal temperatures were recorded at one-minute intervals. Microwaves enhance extraction by generating rapid internal heating, which improves solvent penetration and compound solubilization (Raman and Gaikar. 2002). The resulting extract was filtered and stored at 5 °C until further analysis. All extractions were performed in triplicate.

### Color and color index analysis

The color characteristics of the extracted oils were evaluated in terms of lightness (L*), redness (a*), and yellowness (b*) using a HunterLab ColorFlex EZ Spectrophotometer. The L* value represents lightness on a scale from 0 (black) to 100 (white), while a* and b* denote chromaticity coordinates—positive a* indicates red and negative values indicate green; positive b* indicates yellow and negative values indicate blue. Each measurement was repeated five times to ensure precision. Chroma, indicating color saturation, was calculated using Eq. 1 (Ramos-Escudero et al. 2019):

1 $$Chroma{\rm{ }} = {\rm{ }}{\left( {a{*^2} + {\rm{ }}b{*^2}} \right)^{1/2}}$$

### Peroxide value determination

For mearsuring the peroxide value (POV), five grams of oil were weighed into a 250 mL Erlenmeyer flask and dissolved in 300 mL of an acetic acid/chloroform (3:2, v/v) mixture. Following the addition of 0.5 mL of saturated potassium iodide, the solution was allowed to stand in the dark for 1 min. Next, 30 mL of distilled water was added, and the mixture was titrated with 0.01 M sodium thiosulfate until the yellow color disappeared. Subsequently, 0.5 mL of 1% starch indicator was added, and titration continued until the blue color disappeared (AOAC, 1995). Analyses were performed in five replicates. The peroxide value was calculated using Eq. 2:

2 $${\rm{PO}}\,{\rm{ = }}\,\,{{{\rm{S}}\,{\rm{ \times }}\,{\rm{M}}\,{\rm{ \times }}\,{\rm{100}}} \over {\rm{W}}}$$

Where:

S = volume of sodium thiosulfate (mL),

M = molarity of sodium thiosulfate (mol/L),

W = weight of the oil sample (g),

PO = peroxide value (meq O₂/kg oil).

### Bioactive compound extraction

Bioactive compound extraction was conducted according to the method of Capannesi et al. (2000). Briefly, 2.5 g of oil was vortexed with 5 mL of hexane for 1 min. Then, 3 mL of 20% methanol solution was added, followed by centrifugation at 5000 rpm for 5 min. The upper phase was collected and re-extracted twice under the same conditions. Extracts were stored at 25 °C for subsequent analysis.

### Total phenolic content (TPC)

TPC was quantified using a modified Folin–Ciocalteu method as described by Srisook et al. (2020). A 120 µL aliquot of extract was mixed with 700 µL of deionized water and 30 µL of Folin–Ciocalteu reagent. After a 5-minute incubation in darkness, 150 µL of 7.5% sodium carbonate solution was added. The mixture was further incubated for 30 min at room temperature, and absorbance was measured at 760 nm using a microplate reader (BMG LABTECH, Germany). The experiment were determined in five replicates. TPC was expressed as mg gallic acid equivalents per gram dry weight (mg GAE/g DW).

### DPPH radical scavenging activity

DPPH radical scavenging capacity was assessed following Li et al. (2016), using Trolox as the reference antioxidant. A 500 µL oil extract sample was mixed with 500 µL of 1.25 mM DPPH solution and incubated in the dark for 30 min. A 200 µL aliquot of the reaction mixture was then measured at 520 nm. The test were analysed in five replicates. Radical scavenging activity was expressed as mg Trolox equivalents per 100 g oil (mg TE/100 g).

### ABTS•+ radical scavenging activity

The ABTS•+ assay was performed based on Moo-Huchin et al. (2014). The ABTS•+ radical was produced by reacting 10 mL of 7.5 mM ABTS solution with 10 mL of 2.45 mM potassium persulfate and incubating the mixture in the dark for 16 h. A 200 µL oil extract sample was reacted with 800 µL of the ABTS•+ solution, and absorbance was measured at 732 nm. The experiment was repeated five times. The antioxidant activity was expressed as mg TE/100 g oil.

### Ferric reducing antioxidant power (FRAP)

The FRAP assay was conducted following Benzie and Strain (1996). The FRAP reagent was freshly prepared by mixing 10 mM TPTZ in 40 mM HCl, 20 mM FeCl₃, and 300 mM acetate buffer (pH 3.6) in a 1:1:10 ratio. A 500 µL aliquot of this reagent was added to 500 µL of extract and incubated at 37 °C for 10 min. Absorbance was read at 593 nm. The experiment was repeated five times and the results were reported as mg FeSO₄ equivalents per 100 g oil.

### 1,10-Phenanthroline assay

The antioxidant activity via Fe²⁺–1,10-phenanthroline complex formation was evaluated using the procedure from (Minnoti and Aust 1997). In brief, 150 µL of sample was mixed with 250 µL of 0.2% FeCl₃ in methanol, 125 µL of 0.5% 1,10-phenanthroline, and 475 µL methanol. After incubation in the dark for 20 min, absorbance was measured at 510 nm. The experiment was run in 5 replicates and the results were expressed as mg FeSO₄ equivalent per 100 g of oil.

### Determination of total cannabidiol (CBD) and Δ⁹-tetrahydrocannabinol (thc) contents

The total cannabidiol (CBD) and Δ⁹-tetrahydrocannabinol (THC) contents were determined using high-performance liquid chromatography (HPLC) (Agilent 1260 Infinity, USA), following a method adapted from the AOAC Official Method 2018. A reverse-phase C18 column was employed with isocratic elution using a methanol: water mixture (v/v) at a flow rate of 1.0 mL/min. The injection volume was set at 5 µL, and detection was performed at a wavelength of 230 nm. Column temperature was maintained at 50 °C. Each sample was determined in 5 replicates. Cannabinoid content was calculated as follows:

4 $${\rm{Total }}\,{\rm{CBD }}\,{\rm{ = \% }}\,\,{\rm{C}}\,{\rm{B}}\,{\rm{D }}\,{\rm{ + }}\left( {{\rm{0}}{\rm{.877 }} \times {\rm{ \% }}\,{\rm{CBDA}}} \right)$$

5 $${\rm{Total \,THC = \% }}\,{{\rm{D}}^{\rm{9}}}\,{\rm{THC + }}\left( {{\rm{0}}{\rm{.877 }} \times {\rm{ \%\, THCA}}} \right)$$

### Statistical analysis

All analyses were conducted in five replicates. One-way analysis of variance (ANOVA) was used to identify significant differences among treatment means at a 95% confidence level (*p* < 0.05) using SPSS software. Pearson correlation coefficients were computed to assess relationships between color parameters and antioxidant activity. Principal component analysis (PCA) was also performed to identify underlying patterns and reduce dimensionality in multivariate data. The levels of the principal cannabinoids (CBD, CBDA, THC, and THCA) obtained from IE and IUE methods were compared using Student’s t-test.

## Results and discussions

### Color attributes and extraction methods

Table 1 presents the color characteristics (L*, a*, and b* values) of oils extracted from various cannabis plant parts using different oil-based extraction methods. Oils obtained from the root and pure medium-chain triglyceride (MCT) displayed significantly higher L* values, indicating lighter and brighter hues compared to oils extracted from the sugar leaf and leaf. The observed variation in color intensity during the extraction process is largely due to the enhanced release of natural pigments, particularly chlorophylls and carotenoids, from plant tissues such as sugar leaves. Ultrasound-assisted extraction has been shown to effectively disrupt cell structures, thereby facilitating the diffusion of these pigments into the solvent (Kumar et al. 2022).

**Table 1:** Color of extracted oil at different extraction method

Part of cannabis	Extraction method	Average temperature during process (°C)	L*	a*	b*	chroma	
Sugar leaf	UAE	30	1.35±0.16^de^	2.40±0.16^c^	0.44±0.09^d^	2.45±0.14^c^	
	**IE**	134	1.19±0.07^f^	3.49±0.18^b^	0.82±0.18^bc^	3.58±0.20^b^	
	UIE	136	1.92±0.12^a^	3.19±0.09^b^	0.77±0.13^c^	3.29±0.07^b^	
	MAE	136	1.04±0.17^gh^	3.38±0.13^b^	0.73±0.12^c^	3.43±0.15^b^	
Leaf	UAE	30	1.67±0.05^b^	2.21±0.11^c^	0.99±0.14^b^	1.72±0.12^c^	
	**IE**	136	1.43±0.08^de^	4.11±0.17^a^	1.19±0.16^a^	4.27±0.14^a^	
	UIE	136	1.86±0.14^a^	3.63±0.18^b^	0.89±0.16^bc^	3.74±0.15^ab^	
	MAE	136	1.47±0.78^cd^	3.84±0.87^b^	1.03±0.16^b^	3.98±0.23^ab^	
Root	UAE	30	0.92±0.10^h^	0.21±0.09^d^	-0.47±0.07^e^	0.53±0.06^d^	
	**IE**	136	1.12±0.08^fg^	0.03±0.02^d^	-0.64±0.10^ef^	0.67±0.20^d^	
	UIE	136	1.60±0.14^bc^	0.23±0.09^d^	-0.73±0.08^ef^	0.77±0.09^d^	
	MAE	136	1.21±0.23^f^	0.09±0.09^d^	-0.78±0.07^ef^	0.79±0.09^d^	
Pure MCT	UAE	30	1.51±0.14^cd^	0.37±0.11^d^	-0.65±0.15^ef^	0.87±0.06^d^	
	**IE**	138	1.39±0.12^de^	0.41±0.20^d^	-0.63±0.14^ef^	0.81±0.13^d^	
	UIE	136	1.44±0.06^de^	0.28±0.06^d^	-0.56±0.22^ef^	0.63±0.21^d^	
	MAE	136	1.41±0.09^de^	0.23±0.13^d^	-0.61±0.18^ef^	0.65±0.19^d^	

Data are presented as mean ± standard deviation. Different lowercase superscript letters within the same column indicate statistically significant differences (*p* < 0.05). L*, a*, and b* denote lightness, redness, and yellowness, respectively. UAE, IE, UIE, and MAE refer to Ultrasonic-Assisted Extraction, Infusion Extraction, Ultrasonic-Infusion Extraction, and Microwave-Assisted Extraction, respectively

Moreover, the a*, b*, and chroma values increased notably in extracts obtained through advanced techniques such as infusion extraction (IE), ultrasonic-infusion extraction (UIE), and microwave-assisted extraction (MAE). In contrast, a significant reduction in lightness (L*) was observed following UIE treatment. This color shift, typically from light yellow to deep brown, is likely a consequence of ultrasonic cavitation, which disrupts the plant cell wall structure and enhances membrane permeability, thereby facilitating the release of intracellular compounds including pigments such as chlorophylls and carotenoids. The release of these pigments contributes to the darker coloration of the extract. However, it is also important to consider that excessive ultrasonic energy may lead to bubble coalescence and collapse instability, which could reduce extraction efficiency by interfering with cavitation dynamics (Linares and Rojas 2022).

### Phenolic content and peroxide value determination

The present study demonstrated that cannabis oils possess significantly higher total phenolic content (TPC) compared to medium-chain triglyceride (MCT) oil (*p* < 0.05). Among the cannabis-derived oils, sugar leaf oil exhibited the highest TPC, followed by leaf and root oils (Table 2). Notably, the application of heated infusion extraction markedly increased TPC while concurrently reducing the peroxide value (PV), indicative of enhanced oxidative stability. Both IE and UIE enhanced TPC and reduced PV, indicating improved oxidative stability—consistent with Muangrat and Kaikonjanat (2025). In contrast, infusion with MCT oil alone resulted in a significantly elevated PV.

**Table 2:** Phenolics and peroxide value of extracted oil at different extraction method

Part of cannabis	Extraction method	peroxide value (mg O_2_ eq/kg)	Phenolic content (mg GAE/100 g)
Sugar leaf	UAE	0.130±0.017^def^	37.35±0.61^g^
	**IE**	0.276±0.021^c^	125.27±2.54^a^
	UIE	0.195±0.039^cd^	113.56±7.07^b^
	MAE	0.131±0.02^def^	70.93±3.56^e^
Leaf	UAE	0.079±0.012^ef^	14.13±0.39^i^
	**IE**	0.192±0.086^cd^	100.16±4.61^c^
	UIE	0.179±0.068^cde^	93.90±2.55^d^
	MAE	0.146±0.020^de^	54.54±1.69^f^
Root	UAE	0.063±0.017^e^	2.16±0.38^j^
	**IE**	0.055±0.010^f^	54.03±1.99^f^
	UIE	0.080±0.010^ef^	52.78±0.66^f^
	MAE	0.058±0.058^f^	29.89±1.32^h^
Pure MCT	UAE	0.048±0.011^f^	1.05±0.15^j^
	**IE**	3.113±0.022^b^	2.06±0.26^j^
	UIE	3.534±0.453^a^	1.72±0.50^j^
	MAE	0.058±0.024^f^	4.60±0.44^j^

Data are presented as mean ± standard deviation. Different lowercase superscript letters within the same column indicate statistically significant differences (*p* < 0.05). UAE, IE, UIE, and MAE refer to Ultrasonic-Assisted Extraction, Infusion Extraction, Ultrasonic-Infusion Extraction, and Microwave-Assisted Extraction, respectively

Recent studies have highlighted the influence of extraction techniques and solvent polarity on the phenolic content and antioxidant properties of plant-derived oils. For example, Lanzoni et al. (2023) demonstrated that hemp-based products subjected to in vitro digestion maintained high levels of phenolic compounds and exhibited strong antioxidant activity, indicating their potential health benefits. Additionally, Uoonlue and Muangrat (2019) found that the use of polar solvents such as isopropanol in the subcritical extraction of oil from Assam tea seeds (Camellia sinensis var. assamica) yielded significantly higher phenolic content and antioxidant capacity compared to non-polar solvents like hexane.

Moreover, Ahmad et al. (2021) reported that ultrasonic-assisted extraction effectively enhanced the hydrolysis and release of bound phenolic compounds in wild fruits, further emphasizing the role of advanced green extraction technologies in increasing TPC and improving antioxidant potential. These findings support the idea that optimized extraction methods significantly influence the recovery of bioactive compounds and the oxidative stability of plant-based oils.

The observed inverse relationship between TPC and PV suggests that phenolic compounds contribute to the oxidative stability of hemp seed oils. This is supported by Kaikonjanat and Muangrat (2024), who found that supercritical CO₂ extraction under higher temperature and pressure increased TPC and antioxidant activity, while decreasing peroxide values, indicating enhanced oxidative stability.

Muangrat and Kaikonjanat (2025) also investigated the impact of different extraction methods on the physicochemical properties of hemp seed oil. The researchers found that oils extracted using accelerated hexane methods exhibited higher total phenolic content and correspondingly lower peroxide values, indicating reduced lipid oxidation. This inverse relationship suggests that phenolic compounds play a significant role in enhancing the oxidative stability of hemp seed oils.

These findings align with previous research demonstrating the antioxidant properties of phenolic compounds in various plant-based oils. For instance, Judžentienė et al. (2023) reported a wide range of total phenolic content in hemp seed and flower extracts, with root extracts showing a total phenolic content of up to 321.0 mg/L gallic acid equivalent (GAE). Additionally, Ahmed et al. (2019) measured methanol-derived phenolic content from *Cannabis sativa* leaf at 36.42 mg GAE/g. These studies collectively underscore the importance of phenolic compounds in enhancing the antioxidant capacity and shelf-life of hemp seed oils.

### Antioxidant activity assessment

The antioxidant activities of the cannabis oils were evaluated using DPPH, ABTS•⁺, FRAP, and 1,10-phenanthroline assays (Table 3). All cannabis oil samples exhibited significantly higher antioxidant capacities compared to pure medium-chain triglyceride (MCT) oil. Among the tested samples, sugar leaf oil demonstrated the highest antioxidant potential across all assays. These findings are consistent with Andre et al. (2016), who reported that *Cannabis sativa* leaves contain elevated levels of antioxidant-related phytochemicals, including polyphenols and flavonoids, relative to roots and other plant parts.

**Table 3:** Antioxidant activity in term of DPPH, ABTS, FRAP, and 1,10-Phenanthroline complex

Part of cannabis	Extraction method	anti DPPH (mg trolox eq/100 g oil)	anti ABTS (mg trolox eq/100 g oil)	FRAP (mg FeSO_4_ eq/100 g oil)	1,10-Phenanthroline (mg FeSO_4_ eq/100 g oil)	
Sugar leaf	UAE	2.64±0.13^g^	166.14±5.43^c^	204.43±31.95^d^	0.51±0.04^g^	
	**IE**	10.98±1.01^a^	218.57±5.58^a^	1468.76±26.64^a^	2.50±0.30^a^	
	UIE	9.32±0.41^b^	220.70±4.11^a^	1112.79±31.97^b^	2.12±0.05^b^	
	MAE	5.21±0.14^e^	192.30±8.04^b^	843.57±40.95^c^	1.08±0.26^e^	
Leaf	UAE	1.18±0.14^h^	40.75±5.00^g^	63.60±14.64^fg^	0.18±0.03^i^	
	**IE**	8.64±0.28^c^	134.10±10.85^d^	214.27±21.91^d^	1.86±0.14^c^	
	UIE	7.94±0.33^d^	128.37±6.97^e^	196.59±30.98^d^	1.76±0.15^c^	
	MAE	4.55±0.28^f^	46.60±2.01^f^	88.23±7.82^f^	1.02±0.13^e^	
Root	UAE	0.16±0.04^i^	2.01±0.78^i^	33.76±0.87^hi^	0.25±0.02^h^	
	**IE**	4.93±0.09^ef^	40.40±1.69^g^	156.22±0.67^e^	1.60±0.18^d^	
	UIE	4.75±0.30^ef^	38.16±2.28^g^	149.38±3.90^e^	1.48±0.07^d^	
	MAE	2.65±0.19^g^	10.31±0.34^h^	38.07±8.88^gh^	0.70±0.06^f^	
Pure MCT	UAE	0.32±0.06^i^	5.59±0.36^hi^	2.19±0.20^i^	0.04±0.03^i^	
	**IE**	0.36±0.03^i^	5.44±0.18^hi^	6.19±0.99^i^	0.05±0.02^i^	
	UIE	0.31±0.03^i^	6.59±0.49^hi^	7.87±0.56^hi^	0.03±0.01^i^	
	MAE	0.23±0.07^i^	4.91±0.45^hi^	7.81±0.51^hi^	0.06±0.02^i^	

Data are presented as mean ± standard deviation. Different lowercase superscript letters within the same column indicate statistically significant differences (*p* < 0.05). UAE, IE, UIE, and MAE refer to Ultrasonic-Assisted Extraction, Infusion Extraction, Ultrasonic-Infusion Extraction, and Microwave-Assisted Extraction, respectively. DPPH denotes 2,2-diphenyl-1-picrylhydrazyl radical scavenging activity; ABTS refers to 2,2′-azino-bis(3-ethylbenzothiazoline-6-sulfonic acid) radical scavenging activity; and FRAP indicates ferric reducing antioxidant power

The enhanced antioxidant activity observed in cannabis oils is likely attributable to the migration of phytochemicals—particularly phenolic compounds—into the oil phase during extraction. This phenomenon aligns with the findings of Kalinowska et al. (2022), who demonstrated that ethanol-extracted hemp seed oils exhibited robust antioxidant activities, particularly in FRAP and ABTS assays, highlighting the impact of solvent polarity on antioxidant potential.

Moreover, the extraction technique significantly influenced antioxidant performance. Oils obtained via ultrasound-assisted extraction (UAE) exhibited superior antioxidant activities compared to other methods. This improvement is primarily attributed to the mechanical effects of ultrasound, such as acoustic cavitation, which enhances mass transfer and solvent penetration, thereby facilitating the release of bioactive compounds (Dhanani et al. 2017; Chemat et al. 2017). Similarly, Nazir et al. (2024) reported that UAE markedly improved the recovery of phenolic constituents and antioxidant activity in various medicinal plants, underscoring the efficiency of this green extraction technology.

### Correlation analysis

**Fig. 2:**
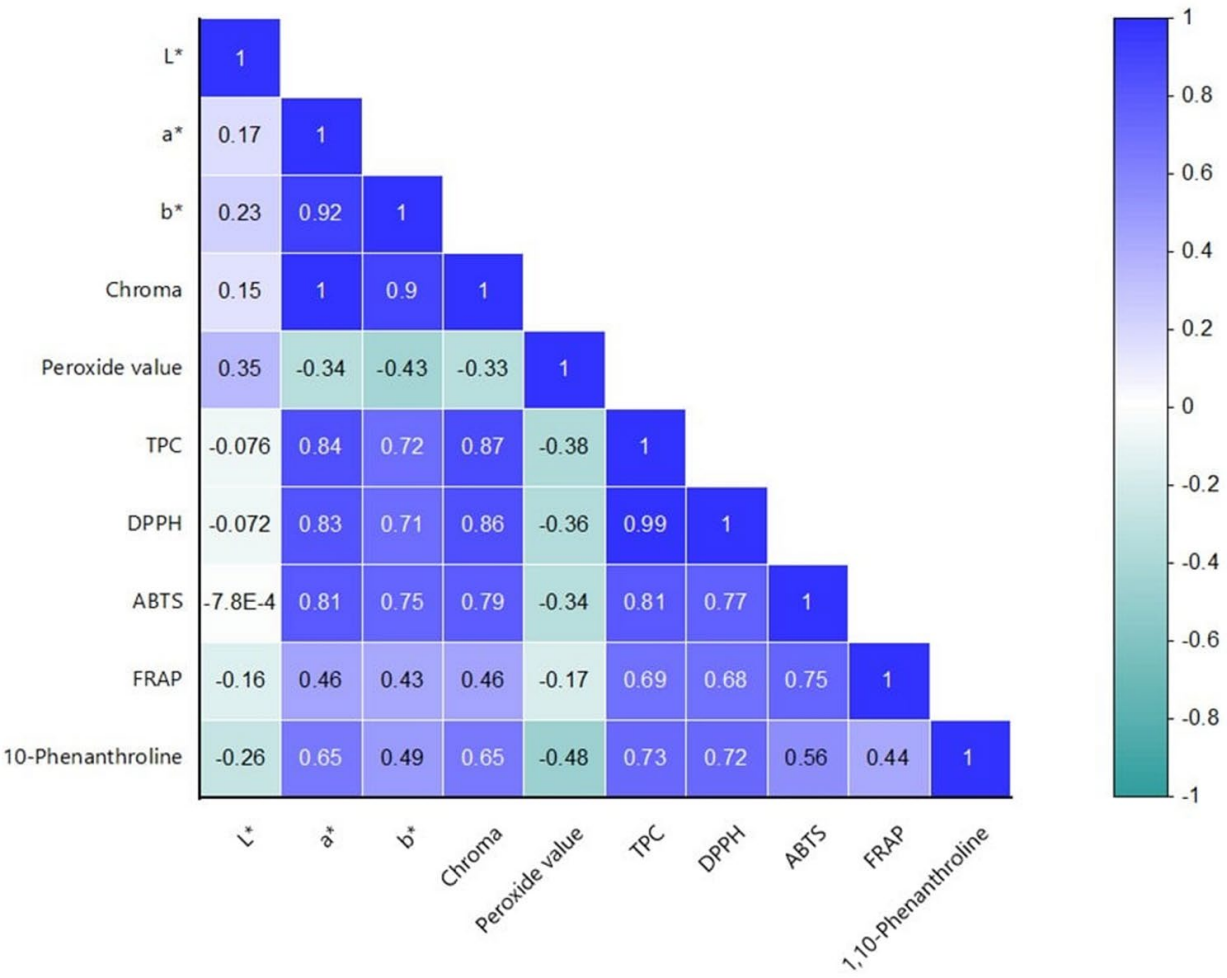
Heatmap correlation of cannabinoid antioxidant properties

Pearson correlation analysis revealed strong positive correlations between color parameters (a* and chroma index) and TPC (R_a*_ = 0.84; R_chroma_ = 0.87), as well as with DPPH (R_a*_ = 0.83; R_chroma_ = 0.86) and ABTS^•+^ (R_a*_ = 0.81; R_chroma_ = 0.79) antioxidant activities (Fig. 2). These findings are in agreement with previous studies by Nguyen et al. (2019) and Garcia et al. (2017), which reported significant correlations between color attributes and antioxidant capacities in medicinal plant extracts.

Moreover, TPC showed strong correlations with DPPH (*R* = 0.99) and ABTS (*R* = 0.81), and moderate-to-strong correlations with FRAP (*R* = 0.69) and 1,10-Phenanthroline (*R* = 0.73) assays. These relationships underscore the pivotal role of phenolic compounds in contributing to the antioxidant potential of cannabis oils.

### Principal component analysis

**Fig. 3:**
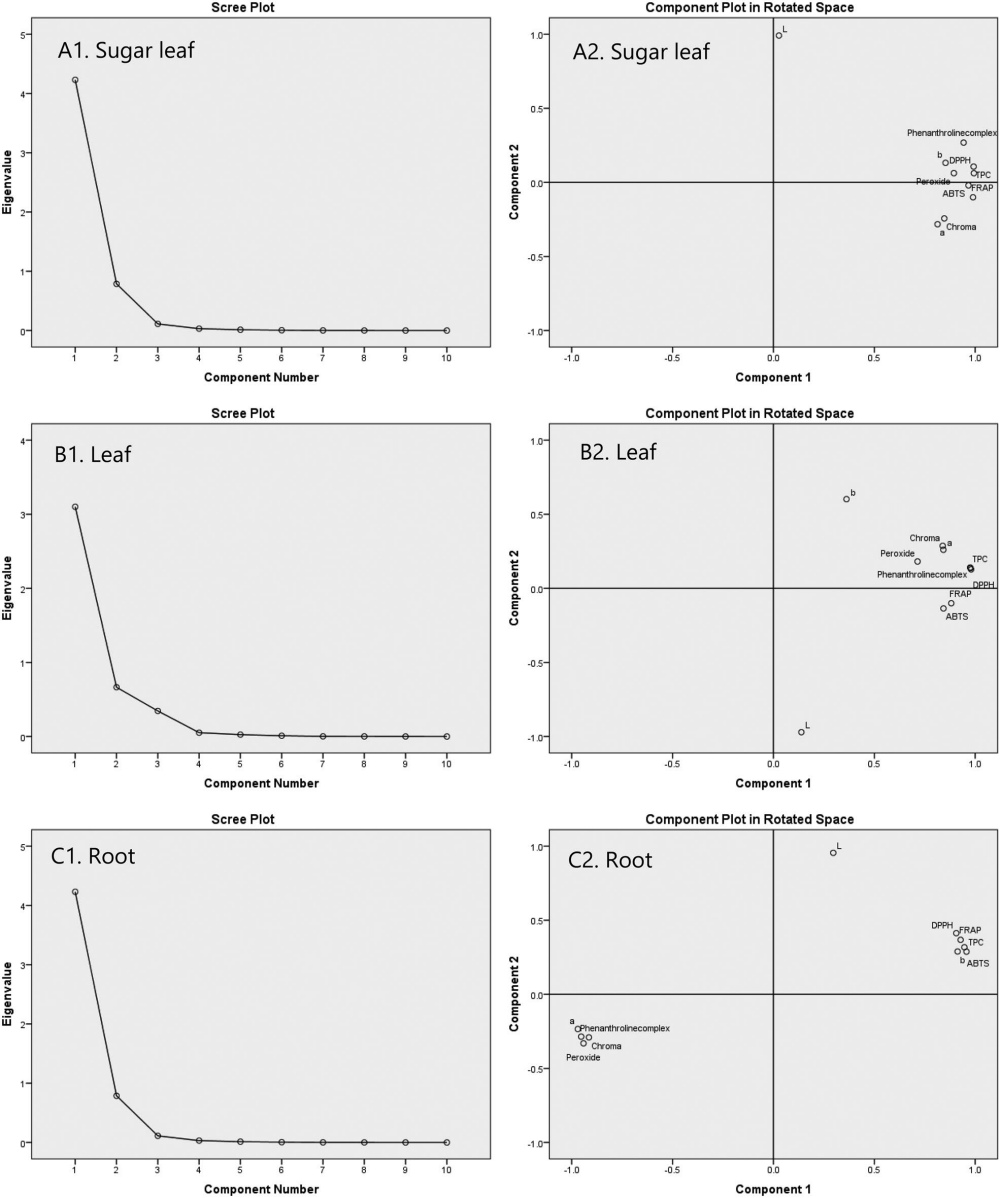
Eigenvalues of each principal component for sugar leaf (**A**1), leaf (**B**1) and root (C1) and biplot (PC1xPC2) of scores and loadings for the principal component analysis (PCA) of (**A**2) sugar leaf, (**B**2) leaf and (**C**2) root

Principal Component Analysis (PCA) was employed to elucidate the relationships among various biochemical and colorimetric parameters of cannabis oils derived from sugar leaves, leaves, and roots (Fig. 3). For sugar leaf oils, the first principal component (PC1) accounted for 81.63% of the total variance, primarily characterized by high positive loadings for TPC, DPPH, and 1,10-Phenanthroline activities. The second principal component (PC2), explaining 15.20% of the variance, was strongly influenced by lightness (L*), indicating a significant inverse relationship between lightness and the yellow-blue dimension (b*).

In leaf oils, PC1 and PC2 explained 73.81% and 15.83% of the total variability, respectively, with PC1 dominated by antioxidant activities and TPC, and PC2 influenced by color parameters. For root oils, PC1 accounted for 79.32% of the variability, characterized by strong positive contributions from TPC and antioxidant activities, and negative contributions from chroma index and peroxide value. These analyses highlight the complex interplay between biochemical constituents and color attributes in determining the quality of cannabis oils.

### Cannabinoid content

High-Performance Liquid Chromatography (HPLC) analysis (Table 4) revealed that oils extracted via IE exhibited higher concentrations of CBD and THC compared to those obtained through UIE. Specifically, IE yielded CBD and THC concentrations of 0.023 g/100 g and 0.721 g/100 g, respectively. These findings are consistent with the study by Xi (2017) which reported that prolonged exposure to elevated temperatures during extraction enhances the recovery of phytochemicals.

**Table 4:** Cannabis hemp sugar leaf extracted by IE and UIE

Extraction method	CBD (g/100 g DM)	CBDA (g/100 g DM)	THC (g/100 g DM)	THC-A (g/100 g DM)	Total CBD (g/100 g DM)	Total $\:{}^{9}$-THC (g/100 g DM)
IE	0.023*	0.004	0.721*	0.029*	0.027**	0.746**
UIE	0.019*	0.003	0.706*	0.031*	0.022**	0.733**

Data are expressed as mean. The * within the same column means statistically significant differences (*p* < 0.05). The ** within the same column denote statistically significant differences (*p* < 0.001). IE and UIE refer to Infusion Extraction and Ultrasonic-Infusion Extraction, respectively. CBD, CBDA, THC, and THCA represent cannabidiol, cannabidiolic acid, Δ⁹-tetrahydrocannabinol, and Δ⁹-tetrahydrocannabinolic acid, respectively. DM denotes dry matter

However, it is important to note that excessive thermal exposure can potentially degrade sensitive compounds. This finding is consistent with Vuong et al. (2011, 2013), who highlighted the importance of optimizing extraction parameters to achieve a balance between extraction yield and the stability of bioactive compounds. Their studies indicated that extended exposure to elevated temperatures may adversely affect phytochemical stability. 

Comparative analysis with previous studies indicates that the IE method yields higher cannabinoid concentrations than other oil-based extraction methods. For instance, Muhammad Zen et al. (2023) reported lower CBD and THC concentrations using a heating and frying method with coconut oil. Similarly, De Vita et al. (2019) observed lower cannabinoid concentrations in olive oil extracts obtained via ultrasonic extraction. These comparisons underscore the efficacy of the IE method in maximizing cannabinoid recovery.

Furthermore, the HPLC data indicated significant decarboxylation of cannabinoid acids (CBDA to CBD and THCA to THC) during the extraction process, facilitated by thermal conditions. This chemical transformation enhances the bioavailability and therapeutic potential of the cannabinoids (Pattnaik et al. 2022).

## Conclusions

In conclusion, this study investigated the effects of different oil-based extraction methods on the antioxidant properties and CBD/THC content of cannabis derived from sugar leaf, leaf, and root. The findings illustrate significant impacts of extraction methods on the color attributes, peroxide value, phenolic content, antioxidant activity, and cannabinoid concentrations in the extracted oils. Specifically, the infusion extraction (IE) method demonstrated superior antioxidant activity compared to alternative methods. Furthermore, IE resulted in elevated levels of total phenolic content and lower peroxide values, indicating enhanced preservation of oil quality. Importantly, the IE method yielded higher concentrations of CBD and THC in sugar leaf oil relative to other extraction techniques. Overall, this research underscores the critical importance of optimizing extraction methods to maximize the nutritional and bioactive properties of cannabis oil. These findings hold promising implications for the development of cannabis-based products tailored for both medical and nutritional applications.

## Data Availability

No datasets were generated or analysed during the current study.

## Abbreviations

MCT: Coconut medium-chain triglycerides
UAE: Ultrasound-assisted extraction
IE: Heated infusion extraction
UIE: Ultrasound-assisted infusion extraction
MAE: Microwave-assisted extraction
DM: Dry matter
GAE: Gallic acid equivalent
TE: Trolox equivalent
ABTS: Scavenging activity
FRAP: Ferric reducing ability power
L*: Lightness
a*: Redness
b*: Yellowness
PO: The oil peroxide
DPPH: 2,2-diphenyl-1-picrylhydrazyl) radical activity
TE Tr: lox equivalents
TPTZ: 2,4,6-tripyridyl-s-triazine
